# How Different Stocking Densities Affect Growth and Stress Status of *Acipenser baerii* Early Stage Larvae

**DOI:** 10.3390/ani10081289

**Published:** 2020-07-28

**Authors:** Lucia Aidos, Alessandra Cafiso, Valentina Serra, Mauro Vasconi, Daniela Bertotto, Chiara Bazzocchi, Giuseppe Radaelli, Alessia Di Giancamillo

**Affiliations:** 1 Department of Veterinary Medicine, University of Milan, 20122 Milano, Italy; marialucia.matelaaidos@unimi.it (L.A.); alessandra.cafiso@unimi.it (A.C.); valentina.serra@unimi.it (V.S.); mauro.vasconi@unimi.it (M.V.); chiara.bazzocchi@unimi.it (C.B.); 2 Department of Comparative Biomedicine and Food Science, University of Padua, 35122 Padova, Italy; daniela.bertotto@unipd.it (D.B.); giuseppe.radaelli@unipd.it (G.R.); 3 Pediatric Clinical Research Center “Romeo ed Enrica Invernizzi”, University of Milan, 20122 Milano, Italy; 4 Coordinated Research Center “EpiSoMI”, University of Milan, 20122 Milano, Italy

**Keywords:** siberian sturgeon, larvae, stocking density, stress, muscle development

## Abstract

**Simple Summary:**

Siberian sturgeon is a freshwater fish species currently at risk of extinction. Few studies have been performed on the environmental conditions during early larval stages, a phase of development where mortality rate is still relevant. In this study, we focused on assessing the impact of different rearing densities during the endogenous feeding stage of the Siberian sturgeon on growth, stress status, and muscle development, using a multidisciplinary approach. Results indicate that lower densities seem more advantageous in this stage of development in terms of growth rate and stress levels. However, rearing larvae at such low densities is not economically feasible in commercial hatcheries but it could be interesting, instead, in larvae production for repopulation purposes.

**Abstract:**

In the present study, a multidisciplinary approach was used in order to evaluate growth, muscle development, and stress status in Siberian sturgeon *Acipenser baerii* larvae at schooling (T1) and complete yolk sac absorption (T2), reared at three stocking densities (low, medium, and high). Larvae growth, morphological muscle development, and whole-body cortisol levels were assessed. The expression of genes involved in the growth process (*igf1* and *igf2*), in the myogenesis (*myog*), and in the regulation of cellular stress (*glut1*, *glut2*, *glut4*, and *hsp70*) was analyzed using a quantitative PCR. Larvae reared at lower densities showed a higher Specific Growth Rate and showed a physiological muscle development. Cortisol levels were low and did not differ significantly, both in different time sampling and across densities, suggesting that either the considered densities are not stressors in this species in the early stages of development or the hypothalamus-pituitary-adrenal (HPA) axis is not yet fully mature. Gene expression of *glut1*, *igf1*, and *igf2* showed an up-regulation in both developmental stages at all the rearing densities considered, while *myog* significantly up-regulated at T1 at the highest density. Considering all of the results, it would seem that lower densities should be used in these stages of development, as these showed a higher growth rate, even if it is not economically feasible in commercial hatcheries. Therefore, choosing an intermediate stocking density could be a good compromise between larval performance and economical feasibility.

## 1. Introduction

In aquaculture, stocking density is a key factor in determining the profitability and economic sustainability of a company [1] and farmers often tend to increase it to favor productivity [2], causing, eventually, chronic stress to the animal [3]. In fish farming, stress takes place following physical and physiological changes in the aquatic environment, such as crowding, handling or changes of physical and chemical, and results in neuroendocrine, physiological, and behavioral alteration [4].

The response of fish towards stress factors may allow a homeostatic recovery (adaptive nature) or may have negative effects on survival, growth, immune response, reproductive capabilities, behavior, and general fitness (maladaptive nature) in case of prolonged exposure to stressors [5,6,7].

When subjected to a stress situation, fish produces two subsequent responses: primary and secondary.

These responses are mainly mediated by the activation of two hormonal axes in fish, the sympatho-chromaffin (SC) axis and the hypothalamic-pituitary-interrenal (HPI) axis. The SC axis activates a fast stress response, involving the cardio-respiratory system by increasing ventilatory and heart rates, heart stroke volume, and blood perfusion in gills and muscle, providing glucose supply to critical tissues, with adrenaline being one of the major mediator hormones. An activated HPI axis contributes to the re-organization of resources by increasing the catabolic pathways, supplying glucidic sources, processing fatty acids for energy, and suppressing other high-cost energy and longer-term processes such as those of immune responses, being cortisol the major mediator. By binding to glucocorticoid (GR) or mineralocorticoid (MR) receptors, cortisol regulates neuro immunoendocrine circuitries, elicits stress-induced immunosuppression, and contributes to allostatic imbalances [8].

Particularly during stressful situations, it may also affect developmental processes such as hatching, growth, and metamorphosis [9,10]. Endogenous cortisol production in many cases does not start before hatching [9,11,12,13,14] but, in early stages of development, the HPI axis may be activated by a stress condition. Indeed, studies conducted on salmonids subjected to two types of stress (temperature shock and handling) indicated that the axis could be activated already after hatching, and [13,15,16] found that the HPI of the white sturgeon, *Acipenser transmontanus*, became functional from the third day post-hatch onward. In lake sturgeon, *Acipenser fulvescens*, temperature had a significant influence on the cortisol response during the prolarval stage. In a previous study [17] with Siberian sturgeon, *Acipenser baerii*, the cortisol concentrations significantly increased in larvae reared at higher temperatures but remained low and comparable with no-stress levels observed in other sturgeon species [16,18]. The authors state that these results may suggest that the increase in cortisol levels could be due to the increased metabolic rate caused by the rearing temperature [19] rather than to the stress response.

The secondary response takes place as a consequence of the released stress hormones [5], which cause changes in the chemistry of the blood and tissues, such as an increase of plasma glucose [20,21]. The stress hormones in combination with cortisol control and elevate glucose production in fish through gluconeogenesis and glycogenolysis pathways [22] to deal with the higher energy demand, which allows fish to prepare for the emergency situation [23].

The fish’s response to stressors has an adaptive nature, allowing for homeostatic recovery, but it becomes maladaptive if the stressor perception is prolonged, having adverse effects on survival, growth, immune response, reproductive capabilities, and behavior. High density in fish farming may induce stress response that, in turn, elevates plasma glucose levels. Alterations in the glucose metabolism are frequently the cause for glycemic changes, which indicates that glucose is mobilized as a result of a challenging situation in many fish species, as reviewed by Polakof et al. [24]. Glucose is distributed in various tissues by means of the glucose membrane transporter (GLUT) proteins in order to restore it to normal levels. The isoforms of GLUT proteins have been categorized according to their sequence and their form in: class I (GLUT1, 2, 3, and 4), class II (GLUT5, 7, 9, and 11) and class III (GLUT6, 8, 10, 12, and the myo-inositol transporter HMIT1) [25,26,27]. Among these proteins, some have been investigated in fish. GLUT1 is responsible for the import and export of glucose into red blood cells. The effect of high density has been studied in Nile tilapia, *Oreochromis niloticus*, demonstrating an increase in *glut1* expression, which can be used as a cellular stress biomarker in aquaculture [28]. The *glut2* gene codes for the sodium-independent glucose transporter (GLUT2), and a significant increase in the number of *glut2* mRNA copies in response to acute and chronic hypoxia has been demonstrated in liver of European seabass, *Dicentrachus labrax*, by Terova et al. [29]. GLUT4 in mammals is expressed mainly in the heart, skeletal muscle, and adipose tissue and constitutes the only insulin-sensitive transporter of the class I GLUTs [26]. GLUT4-homologs were identified in brown trout, *Salmo trutta*, (btGLUT4) [30,31] and in coho salmon, *Oncorhynchus kisutch* (GLUT4) [32] and were expressed in skeletal muscle and adipose tissue, among other tissues. There is evidence that the main site of glucose uptake in fish is the skeletal muscle, which constitutes the only tissue where the uptake of glucose showed an increase after a glucose load, as observed in brown trout [33]. It has been shown that the *glut4* expression increases with the progression of the myogenic differentiation process, which involves an increase in insulin-stimulated glucose transport [34]. The response to various stressors is one of the main fish survival and/or adaptive mechanisms and involves protein changes that include the increase in heat-shock protein synthesis (HSPs) [22]. HSPs are a family of highly conserved proteins used to prevent cell damage by interfering with the uncontrolled protein unfolding that occurs under stress. HSPs have been shown to have a relatively short half-life, but they present a long permanence in fish tissue cells [35], demonstrating that they may play an important role in the long-term adaptation of fish to their habitats [36].

Myogenesis is common to all vertebrates and consists of the specification, proliferation, differentiation, migration, and fusion of the mononucleated muscle precursor cells (MPCs) into new multinucleated myofibers. Embryonic development in teleosts is profoundly influenced by the environmental conditions that determine the rate of myogenesis, the distribution of the number and size of muscle fibers, and gene expression [37]. During the larval phases, the plasticity of fish muscles in response to the environment is usually not reversible due to the rapid rate of ontogenetic changes in this developmental period [37]. The growth potential of the larvae could be affected by modifications on the proliferative capacity of myogenic cells. Both the maximum size and the growth rate are, indeed, related to the number of muscle fibers in young fish [38].

There are several genes involved in myogenesis, among which the insulin growth factor gene complex (*igf1* and *igf2*) and myogenin (*myog*) are reliable indicators to follow muscle development and growth.

Farming fish requires knowledge of fast muscle ontogeny and functional anatomy. In the present study, we focused on the Siberian sturgeon, which has been included in the International Union for conservation of Nature (IUCN) red list for endangered species [39], this means it is considered at risk of extinction in the near future. For this reason, the objective of this study was to evaluate muscle growth and stress status in larvae of Siberian sturgeon reared at three different density conditions during the endogenous feeding period. Information on the most suitable rearing density during this delicate stage of development may provide guidelines for commercial hatcheries that may lead to a better larvae production efficiency.

## 2. Materials and Methods

### 2.1. Experimental Set-Up

The experiment was performed in March/April 2017 at the Experimental Animal Research and Application Centre of Lodi of the University of Milan. Fertilized eggs of Siberian sturgeon, provided by the sturgeon farm Società Agricola Naviglio (Mantova, Italy), were transported at 14 °C in water saturated with oxygen, 24 h after fertilization. After a period of acclimatization, the eggs were incubated at 16 °C and, at hatching (which took place five days after fertilization), temperature was gradually increased up to 19 °C. After hatching, larvae were randomly distributed in rectangular, glass tanks (3 replicates per treatment) at three different densities: low density (LD; 30 larvae/liter, which is the density suggested by FAO 2013 for sturgeon species in this stage of development), medium density (MD; 60 larvae/liter), and high density (HD; 120 larvae/liter), until the yolk-sac was fully absorbed (eight days post-hatch, dph). Chosen densities are representative of the protocols currently used in Siberian sturgeon production farms. Throughout the trial, densities were maintained by adjusting the water level, taking into account T1 sampling and daily mortality. Water quality parameters as oxygen, temperature, and pH were measured every day by Hach HQ30D Portable Meter (Hach Lange, Dusseldorf, Germany). Eggs and larvae were exposed to an artificial photoperiod of 12L:12D. Larvae were reared in a recirculating aquaculture unit, comprised of a sand filter, a biological submerged filter, and a UV lamp sterilization unit. Measurements of ammonia, nitrite, and nitrate were performed at the beginning, at hatching, and at the end of the trial by Hach 2800 Portable Spectrophotometer (Hach Lange, Dusseldorf, Germany) and were in accordance with values recommended for Siberian sturgeon. During the whole trial, it was not necessary to supply any kind of exogenous feeding because larvae only used the nourishment of the yolk sac for their maintenance. Sampling time points consisted of important steps of Siberian sturgeon larvae development: hatching (T0), beginning of the schooling phase (T1), and complete yolk-sac absorption phase (T2). The observation of larvae behavior allowed to identify T1, which took place when larvae became benthonic and started to aggregate in shoals. After T1, larvae were daily observed in order to assess the pigment plug evacuation movement towards the anus; T2 took place when larvae showed the pigment plug evacuation. For each sampling time-point, larvae were collected with a glass beaker and euthanized by over-anesthesia with Ethyl 3-Aminobenzoate, Methanesulfonic A (MS222, Sigma-Aldrich, Saint Louis, MO, USA). All procedures performed in studies involving animals were in accordance with the ethical standards of the OPBA committee of the University of Milan (OPBA_22_2017).

### 2.2. Zootechnical Performance

Survival was estimated by daily recording of dead larvae. Moreover, larvae (N = 3 samples at T0; N = 3 samples for three tanks, for 3 treatments, for two time-points, 3 + (3 × 3 × 3 × 2); N = 57 in total) were weighed and measured in order to determine body weight (*BW*) and total length (*TL*), respectively. Growth performance was described at T1 and T2 using the following parameters:

(1) Specific Growth Rate (*SGR*); where *FBW* and *IBW* denote the final and initial body weight, respectively.
  (1) $$\left(SGR\right)=100\times \left(\frac{ln\;FBW-ln\;IBW\;}{Days}\right)$$
(2) Condition Factor (*K*)
  (2) $$\left(K\right)=100\times \frac{BW}{TL^{3}}$$

### 2.3. Micro-Anatomical Analyses: Histology and Immunofluorescence (Actin)

The hematoxylin/eosin (HE) stain was performed for the evaluation of the structural aspects of the developing lateral muscle tissues. Moreover, micro-anatomical analyses were performed for the detection of anti-rabbit skeletal muscle actin antibody (ACTA1) immunofluorescence. Whole larvae were fixed in 4% (*v*/*v*) paraformaldehyde (N = 3 samples at T0; N = 3 samples for three tanks, for 3 treatments, for two time-points, 3 + (3 × 3 × 3 × 2); N = 57 in total). The samples were then dehydrated in a graded 50% (*v*/*v*), 70% (*v*/*v*), 95% (*v*/*v*), and 100% (*v*/*v*) ethanol series, embedded in paraffin and transversally cut into 5-µm-thick serial sections. After rehydration, sections were incubated with the first-step primary antiserum 1:1000 anti-rabbit Skeletal Muscle Actin Antibody (ACTA1, LifeSpan BioSciences LS-B4203, Seattle, WA, USA) for 48 h at 18–20 °C, then washed in PBS (Phosphate-buffered saline) for 10 min and incubated with a solution of 10 µg/mL goat biotinylated anti-rabbit IgG (Vector Laboratories Inc., Burlingame, CA, USA) for 6 h at 18–20 °C.

The sections were then washed twice in PBS and treated with Fluorescein-Avidin D (Vector Laboratories Inc. Burlingame, CA, USA) 10 µg/mL in NaHCO_3_, 0.1 M, pH 8.5, 0.15 M NaCl for 1 h at 18–20 °C. Finally, slides with tissue sections were embedded in Vectashield Mounting Medium with DAPI (4′,6-diamidino-2-phenylindole) (H-1200, Vector Laboratories Inc., Burlingame, CA, USA) and observed using a confocal laser scanning microscope (FluoView FV300; Olympus, Segrate, Italy). The immunofluororeactive structures were excited using Argon/Helio–Neon–Green lasers with excitation and barrier filters set for fluorescein. Images containing superimposition of fluorescence were obtained by sequentially acquiring the image slice of each laser excitation or channel. The specificity tests for the used antibodies were performed by incubating other sections in parallel with: (i) PBS instead of the specific primary antibodies; (ii) PBS instead of the secondary antibodies. The results of these controls were always negative (i.e., staining was abolished).

### 2.4. Cortisol Extraction and Radioimmunoassay (RIA)

Whole-body cortisol analyses were performed in frozen larvae by a specific microtitre radioimmunoassay (RIA) as described by Simontacchi et al. [16]. Larvae were pooled (N = 3 samples at T0; N = 3 samples for three tanks, for 3 treatments, for two time-points, 3 + (3 × 3 × 3 × 2); N = 57 in total), weighed, thawed out, and pulverized in liquid nitrogen, and the resulting powders were suspended in 1 mL PBS (pH 7.2). Subsequently, the suspension was extracted with 8 mL of diethyl ether, and the supernatant was evaporated to dryness. The dry extracts were dissolved in 0.5 mL of PBS and varying aliquots were used for radioimmunoassay (RIA). The entire procedure is detailed in Reference [17].

### 2.5. Gene Identification and Primers Design

Genes involved in cellular stress reactions (*glut1*, *glut2*, *glut4*, and *hsp70*) and genes involved in growth (*igf1* and *igf2*) and myogenesis (*myog*) were selected for further molecular analyses. The *rpl6* (coding for Ribosomal protein L6) and *gapdh* (coding for Glyceraldehyde 3-phosphate dehydrogenase) genes were used as reference [17]. Primer sequences for the amplification of *hsp70*, *igf1*, *myog*, *rpl6*, and *gapdh* gene fragments, annealing temperatures, and the amplification size of each fragment are described by Aidos et al. [17]. Specific primers for the amplification of *glut1*, *glut2*, *glut4*, and *igf2* gene fragments were de novo designed. Briefly, gene sequences from *Acipenser* spp. and some teleostean species were selected in order to perform alignments with the Basic Local Alignment Search Tool (NCBI BLAST), using a previously published assembled transcript of Siberian sturgeon as the reference database [40]. The annealing temperatures and the amplification size of *glut1*, *glut2*, *glut4*, and *igf2* gene fragments are reported in Table 1.

### 2.6. RNA Extraction and cDNA Synthesis

The sampling was performed at T1 and T2. Larvae were immediately stored at −80 °C soon after the sampling procedure. Total RNA was extracted from each frozen larval sample (N = 3 samples for three tanks, for 3 treatments, for two time-points, 3 × 3 × 3 × 2; N = 54 in total) using RNeasy Mini Kit^®^ (Qiagen, Hilden, Germany) and eluted in a final volume of 40 μL of RNase-free water. Double treatment with DNase enzyme was performed in order to remove any genomic DNA contamination, according to manufacturer instructions. Five hundred nanograms of RNA was retro-transcribed to cDNA using Quantitect Reverse Transcription Kit^®^ (Qiagen, Hilden, Germany) following manufacturer protocol. An additional reaction without retrotranscriptase enzyme was performed to verify the complete DNA removal. cDNAs were stored at −80 °C until subsequent use.

### 2.7. Gene Expression Profiles

Gene expression was analyzed by quantitative real-time PCR (qPCR) in larvae collected at the three rearing densities. cDNA samples were used as template in qPCR using a CFX connect Real-Time PCR instrument (Bio-Rad, California, CA, USA) and Universal SYBR^®^ Green Supermix (Bio-Rad, California, CA, USA) as fluorescent molecule. The amplification conditions were: 150 nM (final concentration) of forward and reverse primers; 98 °C for 30 s, 40 cycles of 98 °C for 15 s, 58 °C (*igf2*) or 62 °C (*glu1*, *glut2*, *glut4*) for 30 s; and a melting profile was included after the last amplification cycle. Annealing temperatures were defined according to primers melting temperatures indicated in Aidos et al. [17] and in Table 1. Cycle threshold (Ct) values were determined for each gene and normalized using *rpl6* and *gapdh* genes as reference. The results of MD and HD relative expression of each gene at T1 and T2 (calculated using the ΔΔCt method) were compared to the ones of LD, considered as calibrator.

The amplified *glut1*, *glut2*, *glut4*, and *igf2* gene fragments were loaded on agarose gel, purified, and Sanger-sequenced in order to confirm the specificity of the amplification. The obtained sequences were deposited in GenBank.

### 2.8. Statistical Analysis

Statistical analysis of the data was performed using the 2-way ANOVA with densities and developmental stages as main factors of the Statistical Analysis Software (SAS version 8.1, Cary Inc., Rural Hall, NC, USA). Each rearing tank was considered as the individual value. The data were presented as least squared means SEM (standard error of the mean). Differences were considered significant at *p* < 0.05 and highly significant at *p* < 0.01.

## 3. Results

This section may be divided by subheadings. It should provide a concise and precise description of the experimental results, their interpretation as well as the experimental conclusions that can be drawn.

### 3.1. Water Parameters

The level of oxygen dissolved in the water was maintained up to the FAO suggested value (6 mg/L) described for this species during the larval stage for all treatments (FAO, 2013). Values of pH were between 8.5 and 8.8 throughout the trial, which fall within the physiological range described for this species in this developmental phase. Total ammonia and nitrites were below 0.05 mg/L throughout the trial.

### 3.2. Larval Mortality, Development, and Growth

From T1 to T2, mortality significantly decreased (Table 2; *p* < 0.01); within T1, the LD group showed lower mortality than both MD and HD groups (Table 2; *p* < 0.05). At T2 no differences were found between treatments (Table 2).

Siberian sturgeon larvae development at T1 and T2 occurred, respectively, at 5 dph and 8 dph and was thus not affected by rearing densities. Data on growth are reported in Table 3. Both body weight and total length of larvae reared at LD were higher at T2 compared to the other two densities. Larval growth expressed as SGR was significantly improved at T2 phase for larvae reared at LD (*p* < 0.05). Additionally, K factor apparently was not significantly affected among larvae by different densities at both T1 and T2.

### 3.3. Micro-Anatomical Analyses: Histology and Immunofluorescence (Actin)

**Figure 1:**
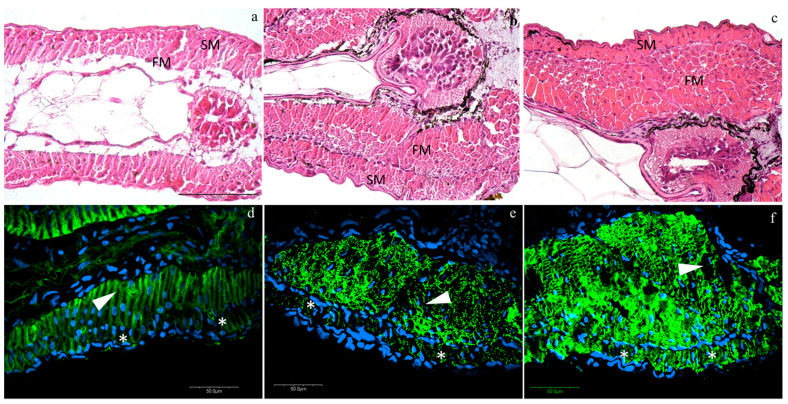
Representative images of hematoxylin/eosin (HE) (**a**–**c**) and actin immunofluorescence (**d**–**f**) at different rearing densities at different time-points: (**a**,**d**) at T0; (**b**,**e**) at low density (LD) at T1; (**c**,**f**) at medium density (MD) at T2. SM, slow muscle cells; FM, fast muscle cells. Green staining, anti-rabbit skeletal muscle actin antibody (ACTA1) immunofluorescence; blue staining, nuclei; asterisks (*), slow muscle; arrow-heads, fast muscle. HE scale bar is located in figure a and refers to 100 µm; actin immunofluorescence scale bars are located in each figure.

Histological analyses revealed an anatomically regular muscle development. At T0, larvae presented an external monolayer of slow muscle cells (SM), as well as an internal monolayer of fast muscle cells (FM) (Figure 1a). Both layers of SM and FM showed a considerable expansion into multilayers from T0 to T1 and from T1 to T2, especially the FM (Figure 1b,c). ACTA1 is the predominant actin isoform in postnatal skeletal muscles: the interaction between skeletal muscle α-actin and the various myosin heavy chain proteins in the different muscle fiber types generates the force of muscle contraction. Skeletal muscle α-alpha actin is thus of fundamental importance to normal muscle contraction and for this reason, we used it to follow the muscle fiber development and organization. ACTA1 immunofluorescence was detected in the cytoplasm of muscle cells (green staining, Figure 1) in all developmental stages. Moreover, no morphological differences among densities were detected in the muscle structure at all stages observing a normal actin myofibrillar organization in both fast and slow-twitch muscle: at T0, slow and fast muscle showed a monolayer (Figure 1a, blue staining, nuclei; asterisks, slow muscle; arrow-heads, fast muscles), while at T1 and T2, the fast muscle expanded in multilayers, whereas the slow muscle still presented a monolayer structure (Figure 1b,c, respectively).

### 3.4. Whole-Body Cortisol

**Figure 2:**
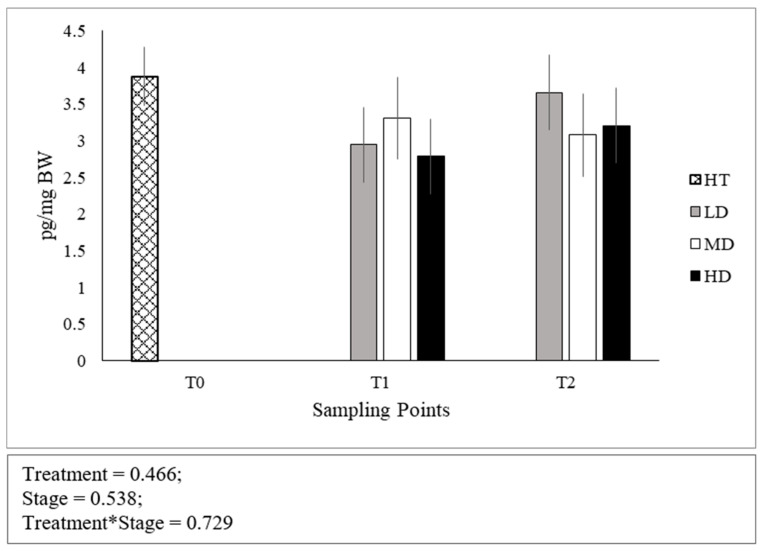
Whole-body cortisol concentrations expressed as pg per mg of body weight. Error bars indicate the standard error of the mean for each treatment/stage of development.

No significant differences were found in the cortisol levels between stages of development (Figure 2). Both at T1 and T2, no differences were found across densities and the interaction between developmental stage and density was not significant.

### 3.5. Stress and Growth-Related Gene Expressions

**Figure 3:**
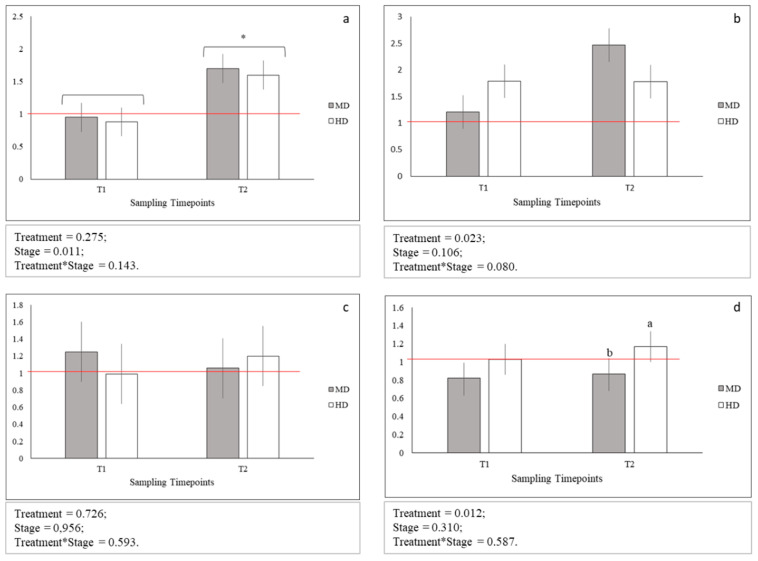
Relative gene expression of: (**a**) *glut1*; (**b**) *glut2*; (**c**) *glut4*, and (**d**) *hsp70*; Means with asterisks differ significantly between stages of development (*p* < 0.05); ^a,b^ Means with different superscripts differ significantly between treatments (*p* < 0.05); red line indicates the up- and down-regulation limit.

**Figure 4:**
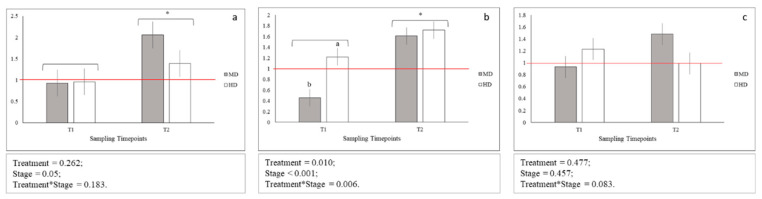
Relative gene expression of: (**a**) *igf1*; (**b**) *igf2*; (**c**) *myog*; Means with asterisks differ significantly between stages of development (*p* < 0.05); ^A,B^ Means with different superscripts differ significantly between treatments (*p* < 0.01); ^a,b^ Means with different superscripts differ significantly between treatments (*p* < 0.05); red line indicates the up- and down-regulation limit.

Sequences obtained from the *glut1*, *glut2*, *glut4*, and *igf2* gene fragments were deposited in GenBank under the accession numbers MN711652-MN711654 and MN733733 (accession numbers of *hsp70*, *igf1*, *myog*, *rpl6*, and *gapdh* gene fragments were previously reported in Reference [17]). The results of relative expressions of the stress- (*glut1*, *glut2* and *glut4*, and *hsp70*) and growth-related (*igf1*, *igf2*, and *myog*) genes are presented, respectively, in Figure 3 and Figure 4. Briefly, the results of genes relative expression (calculated using the ΔΔCt method) for MD and HD samples at T1 and T2 were compared to ones of LD, considered as calibrator. In addition, since for LD treatment no significant differences were observed between absolute expression values of the analyzed genes at both T1 and T2, results showed in Figure 3 and Figure 4 can be compared both across treatments and between time-points (data shown in Table S1).

No statistical differences between *glut1*, *glut2*, and glut4 gene expression levels across MD and HD were observed both at T1 and T2 (Figure 3 a–c). On the contrary, the expression level of *hsp70* gene at HD was observed significantly increased only at T2 (*p* < 0.05; Figure 3d). In addition, *glut1* gene at MD and HD was significantly more expressed at T2 when compared to T1 (*p* < 0.05 Figure 3a). Finally, the expression levels across MD and HD at both T1 and T2 for *glut4* and *hsp70* genes and at T1 for *glut1* and *glut2* genes were comparable to those observed for LD samples. An up-regulation was instead observed in the expression level of *glut1* gene for both MD (*p* < 0.05) and HD (*p* < 0.05) at T2, and of *glut2* gene only for MD at T2 (*p* < 0.05).

No statistical differences between *igf1* gene expression levels across MD and HD were observed both at T1 and T2, while statistical differences between *igf2* gene expression levels across MD and HD were observed only at T1 (Figure 4b; *p* < 0.05). In addition, *igf1* and *igf2* genes were significantly more expressed at T2 when compared to T1 (Figure 4a,b; *p* < 0.05 and *p* < 0.001, respectively). Moreover, *igf1* and *igf2* genes showed an upregulation at T2 for MD (*igf1*; *p* < 0.05) and for both MD and HD (*igf2*; *p* < 0.05) when compared to LD samples. Additionally, *igf2* gene downregulated at T1 for MD when compared to LD (*p* < 0.05). No statistical difference across time-points and treatment was observed for *myog* gene expression levels (Figure 4c).

## 4. Discussion

Siberian sturgeon is an endangered species, which began to undergo a sharp decline in the 1930s (years in which the demand was particularly higher) and continues to decline nowadays. The success and diffusion of Siberian sturgeon farming are due to the great rusticity, the reduced oxygen demand already in the juvenile phase, and the high quality of the derived products (caviar, meat, and skin) [41]. However, the early larval stages still present relevant mortalities, and little studies have been performed concerning the effects of the environmental factors. Understanding the mechanisms that underlie the development and growth of muscle and the state of stress in the early life stages is, therefore, essential in order to identify optimal strategies for rearing and conservation of Siberian sturgeon. For this purpose, in this study, the effect of different rearing densities in early larval stages of Siberian sturgeon was analyzed in terms of early muscular development and growth, as well as stress status, using a multidisciplinary approach.

Stocking density is an important environmental factor that has a significant impact on fish rearing. Many studies indicate that high rearing density increases stress and, at the same time, induces the inhibition of growth in fish [5,42,43]. Some authors suggest that the negative influence of high stocking densities on growth is due to a deterioration of water conditions [43]. In this study, water quality parameters were deliberately maintained stable, thus eliminating the effect of water quality on the growth performance of the larvae. Significant differences in growth were found among densities. Indeed, some authors report that sturgeons are quite sensitive to high rearing densities: in juvenile Atlantic sturgeon, *Acipenser oxyrinchus*, an increased rearing density resulted in a weight reduction [44,45]; similar results have been obtained by Reference [1] in juveniles of beluga, *Huso huso*. Furthermore, in our study, actually, larvae reared at a low stocking density presented a significantly higher body weight, length, and SGR than those reared at medium and high densities. On the contrary, Reference [46] reported that high rearing densities had no impact on the final weight of juvenile lake sturgeon [47]. Instead, in a study conducted with Persian sturgeon, *Acipenser persicus*, beluga, and Stellate sturgeon, *Acipenser stellatus*, concluded that during the endogenous feeding phase, growth is independent of stocking density. It would seem, therefore, that species are differently sensitive to stocking densities, both in terms of growth and stress response. To date, it has been shown that a high rearing density constitutes an environmental stress factor for adult sturgeons [48]. In adult Atlantic cod (*Gadus morhua*) [49], it was observed that short-term overcrowding leads to a temporary rise of plasma cortisol and an increase in the expression levels of stress-related genes. In our study, however, whole-body cortisol level did not change in larvae exposed to different stocking densities, suggesting that this factor is not a stressor in *A. baerii*’s early stages of development, or that the HPA axis is not yet fully mature. In lake sturgeon, Reference [50] assessed the impact of the rearing environment during early larval stages and observed an increase in cortisol production from hatching to the beginning of the exogenous feeding in non-stressed larvae and a delay in the HPI axis development in the stressed ones. Moreover, a study performed by our group [17], where the effect of different rearing temperatures was assessed in Siberian sturgeon larvae, suggested that larvae were not able to produce a stress response in terms of cortisol production. The development of the HPI axis is known to occur at different times among species, for instance, before or after hatching, or close to metamorphosis [9,13,14,16,51,52]. For this reason, we suggest that the poor stress-coping ability observed in our study protects larvae from the elevated metabolic demands involved by stress responses; moreover, it seems to promote growth and survival as also observed by Reference [53] in Ballan wrasse, *Labrus bergylta*. The expression of stress-related genes indicates a negative effect of MD and HD at the end of the trial and this is in agreement with a study conducted by Reference [54] on European seabass, in which an increase in the expression levels of the *glut2* gene under stress conditions due to high stocking densities was detected. Different densities, however, did not affect the expression of *glut4* gene. Caipang et al. [49] studied the effect of short-term overcrowding in Atlantic cod and found that *glut4* gene was significantly upregulated, indicating its possible participation in the stress response of this fish species by aiding the metabolic requirements [24] that glucose transporters (GLUTs) are reported in numerous fish tissues including insulin-sensitive GLUT4 homolog in muscle tissue, yet muscle tissue remains a poor glucose user and this could be the reason for our results. Furthermore, several authors have concluded that there are important inter-individual responses to high density [55,56].

In our study, the expression of *hsp70* was higher at HD when compared with MD. Moreover, in Senegalese sole, *Solea senegalensis* [56], and European seabass [57,58], an increase of the mRNA levels of *hsp70* was observed after crowding stress. Reference [59] observed that the *hsp70* gene expression was significantly density-dependent, being up-regulated in higher densities in rainbow trout, *Oncorhynchus mykiss* juveniles.

The skeletal muscle morphology at all stages at different rearing conditions was evaluated since stocking density can also affect muscle development. Actin is a cytoskeletal protein that exerts a broad range of functions in almost all eukaryotic cells such as maintenance of the cytoskeleton, cell motility, and muscle contraction. Previous studies revealed that ACTA1 is expressed in skeletal muscle of fish [60]. In our study, ACTA1 immunofluorescence was used to follow morphological development of the skeletal muscle in Siberian sturgeon, together with HE staining. Considering different stages, it can be noticed that already at T0 it was possible to observe the difference between slow fibers and fast fibers. The slow fibers appeared as a monolayer from the time of hatching until the whole yolk sac was absorbed. In contrast, the fast fibers showed expansion from T0 up to T2. Therefore, muscle development appeared regular for all studied rearing densities, as also observed by Reference [61].

As for growth-related genes expression, overall, it seems that high densities (particularly MD) lead to a higher expression of both *igfs* and *myog* at T2 when compared to T1. Reference [62] observed that Siberian sturgeon larvae subjected to LD and MD presented higher muscle fibers proliferation at the end of the trial, as well as a higher area of polygonal fast fibers, when compared to HD. These results are consistent with what was obtained in the present study, taking into account that IGFs in fish stimulate myogenic cells proliferation and differentiation [63,64] and that the gene *myog* is a marker of myogenic cell recruitment for stratified hyperplasia in teleosts [65]. It would seem, thus, that higher stocking densities have a positive effect on growth (especially MD), even if this was not observed in terms of body weight, length, and SGR in this short period. Indeed, larvae reared at LD presented higher zootechnical parameters when compared to MD and HD, as already discussed. The higher expression of growth-related genes in larvae subjected to MD and HD may indicate a higher growth potential in these groups, which was not phenotypically observed by the end of the experiment. High stocking densities could lead to a stress situation with the following increase of energy needs and utilization of energy reserves.

## 5. Conclusions

For a better interpretation of the influence exerted by the rearing density on stress and muscle growth of Siberian sturgeon, it would seem necessary to evaluate the expression levels of other genes involved in the same regulation. Subjecting larvae to a concrete stress factor could also allow to verify if the stress response is active in this early stage of development. Furthermore, performing assays at intermediate time-points could establish the precise gene expression modulation in terms of stage of development and stocking density.

Lower stocking densities seem to be the most favorable ones in this stage of development, as already reported. However, in commercial hatcheries, rearing larvae in such low densities is not economically feasible. Therefore, choosing an intermediate stocking density could be a good compromise between larval performance and economic feasibility. On the opposite, when the production purpose is natural populations restoring, rearing pre-larvae in very low densities may be important in increasing the successful introduction of larvae in their natural habitat.

Finally, as during the endogenous feeding phase we had no clear results regarding rearing density related stress, it would be interesting to assess the effect of density during the exogenous feeding phase, in which larvae may present a stronger stress response.

## Figures and Tables

**Table 1:** *glut1*, *glut2*, *glut4*, and *igf2* primer sequences, annealing temperatures, and amplification size of each fragment.

Genes	Primer Forward (5′–3′)	Primer Reverse (5′–3′)	Tm F	Tm R	Fragment Size (bp)
*igf2*	GCTGAAACGCTATGTGGTG	GTGACCTTCGGATGTTTG	59	60	109
*glut1*	AGCCCATTCCTCCAACCTC	GAGTTTCGCCTCCCAAAGC	62	62	124
*glut2*	CTATCGTGGTGCCTTGGGA	GCCCCTGACAAGCCCAGAA	62	64	132
*glut4*	GGCAGCCCATCATCATCGCC	CCACGCCCGCCTTCTCAAAG	63	63	105

**Table 2:** Cumulative mortality rate (%) of Siberian sturgeon larvae reared at different densities at T1 (from T0 to T1) and T2 (from T1 to T2).

	Low-Density	Mid-Density	High-Density
**T1**	29.83% ± 1.66 ^a^	36.50% ± 1.86 ^b^	35.66% ± 2.35 ^b^
**T2**	0.66% ± 0.35	2.0% ± 0.88	3.3% ± 2.3

Within a row, means with different superscript letters differ significantly (*p* < 0.05). Absence of superscript indicates no significant difference between treatments. Values are means ± Standard error. Treatment < 0.01; Stage < 0.01; Treatment × Stage < 0.01.

**Table 3:** Growth performance of Siberian sturgeon larvae reared at different densities in the time-points T0, T1, and T2.

	Growth Parameters	T0	Low-Density	Mid-Density	High-Density
**T0**	Body Weight mg	12.65 ± 1.56			
	Total Length mm	10.5 ± 0.17			
	Condition Factor	1.13 ± 0.05			
**T1**	Body Weight mg		24.29 ± 0.92	24.08 ± 0.98	23.80 ± 0.95
	Total Length mm		15.35 ± 0.10	15.07 ± 0.18	15.33 ± 0.20
	Condition Factor		0.67 ± 0.03	0.71 ± 0.03	0.67 ± 0.03
	Specific Growth Rate		5.58 ± 0.28	5.51 ± 0.30	5.43 ± 0.30
**T2**	Body Weight mg		33.01 ± 0.87 ^a^	30.31 ± 0.74 ^b^	30.15 ± 0.87 ^b^
	Total Length mm		17.73 ± 0.13 ^a^	16.96 ± 0.13 ^b^	17.11 ± 0.16 ^b^
	Condition Factor		0.59 ± 0.03	0.62 ± 0.02	0.61 ± 0.03
	Specific Growth Rate		3.72 ± 0.10 ^a^	3.27 ± 0.11 ^b^	3.36 ± 0.17 ^b^

Within a row means with different superscript letter differ significantly (*p* < 0.05). Absence of superscript indicates no significant difference between treatments. Values are means ± Standard error. Body Weight: Treatment < 0.01; Stage < 0.01; Treatment × Stage = 0.772. Total Length: Treatment < 0.01; Stage < 0.01; Treatment × Stage = 0.373. SGR: Treatment = 0.243; Stage < 0.01; Treatment × Stage = 0.817. K: Treatment < 0.01; Stage = 0.003; Treatment × Stage = 0.844.

## Supplementary Materials

The following are available online at https://www.mdpi.com/2076-2615/10/8/1289/s1, Table S1: LD gene expression raw data.
