# Supplementary material for: How Different Stocking Densities Affect Growth and Stress Status of Acipenser baerii Early Stage Larvae
Source: Animals (Basel). 2020 Jul 28;10(8):1289. doi: 10.3390/ani10081289 (PMC7460203; doi:10.3390/ani10081289)
Supplement: Supplementary file 1 [file animals-10-01289-s001.pdf]

animals

MDPI

| *igf1* values | | | *igf2* values | | | *glut1* values | | | *glut2* values | | | *hsp70* values | | | *myog* values | | |
|---|---|---|---|---|---|---|---|---|---|---|---|---|---|---|---|---|---|
| *gapdh /rpl6* Ct mean | *igf1* Ct | ΔCt (Delta Ct) | *gapdh /rpl6* Ct mean | *igf2* Ct | ΔCt (Delta Ct) | *gapdh /rpl6* Ct mean | *glut1* Ct | ΔCt (Delta Ct) | *gapdh /rpl6* Ct mean | *glut2* Ct | ΔCt (Delta Ct) | *gapdh /rpl6* Ct mean | *hsp70* Ct | ΔCt (Delta Ct) | *gapdh /rpl6* Ct mean | *myog* Ct | ΔCt (Delta Ct) |
| 17.58 | 28.41 | 10.84 | 17.50 | 24.22 | 6.72 | 17.58 | 23.59 | 6.02 | 17.58 | 24.62 | 7.05 | 17.50 | 16.15 | -1.35 | 17.50 | 23.80 | 6.30 |
| 17.21 | 28.77 | 11.57 | 17.21 | 24.59 | 7.39 | 17.21 | 24.06 | 6.86 | 17.21 | 23.97 | 6.77 | 17.21 | 16.54 | -0.66 | 17.21 | 23.49 | 6.29 |
| 17.77 | 28.85 | 11.09 | 17.75 | 25.65 | 7.90 | 17.77 | 24.22 | 6.46 | 17.83 | 25.17 | 7.34 | 17.75 | 16.54 | -1.21 | 17.75 | 23.87 | 6.12 |
| 17.38 | 28.12 | 10.74 | 17.38 | 24.73 | 7.35 | 17.38 | 22.93 | 5.55 | 17.38 | 25.14 | 7.76 | 17.38 | 15.18 | -2.20 | 17.38 | 23.31 | 5.93 |
| 17.68 | 29.07 | 11.40 | 17.58 | 26.56 | 8.98 | 17.56 | 23.65 | 6.10 | 17.68 | 27.44 | 9.77 | 17.58 | 16.57 | -1.01 | 17.58 | 23.74 | 6.16 |
| 18.52 | 30.15 | 11.64 | 18.52 | 26.38 | 7.87 | 17.85 | 25.18 | 7.33 | 18.52 | 26.62 | 8.11 | 18.52 | 17.16 | -1.36 | 18.52 | 24.92 | 6.41 |
| 16.72 | 27.44 | 10.73 | 17.23 | 22.93 | 5.70 | 16.72 | 22.16 | 5.45 | 16.72 | 23.50 | 6.79 | 17.23 | 16.05 | -1.18 | 17.23 | 23.27 | 6.04 |
| 17.71 | 29.18 | 11.47 | 17.71 | 24.50 | 6.79 | 17.71 | 24.90 | 7.19 | 17.71 | 25.07 | 7.36 | 17.71 | 16.32 | -1.39 | 17.71 | 24.28 | 6.57 |
| 17.35 | 29.73 | 12.39 | 16.92 | 25.57 | 8.66 | 17.35 | 26.40 | 9.06 | 16.39 | 23.98 | 7.60 | 16.92 | 17.45 | 0.54 | 16.39 | 22.45 | 6.07 |
| 16.42 | 27.80 | 11.39 | 16.42 | 23.45 | 7.04 | 16.42 | 23.04 | 6.63 | 16.38 | 23.32 | 6.94 | 16.42 | 15.45 | -0.97 | 16.40 | 21.97 | 5.58 |
| 16.73 | 28.85 | 12.12 | 16.75 | 24.23 | 7.49 | 16.73 | 24.21 | 7.48 | 16.73 | 25.42 | 8.69 | 16.75 | 16.21 | -0.54 | 16.75 | 25.16 | 8.42 |
| 16.59 | 29.24 | 12.66 | 16.59 | 24.10 | 7.52 | 16.59 | 24.15 | 7.57 | 16.59 | 24.56 | 7.98 | 16.59 | 17.26 | 0.68 | 16.59 | 23.89 | 7.31 |

**Table S1.** LD gene expression raw data.
